# Eschar-associated Spotted Fever Rickettsiosis, Bahia, Brazil

**DOI:** 10.3201/eid1702.100859

**Published:** 2011-02

**Authors:** Nanci Silva, Marina E. Eremeeva, Tatiana Rozental, Guilherme S. Ribeiro, Christopher D. Paddock, Eduardo Antonio G. Ramos, Alexsandra R.M. Favacho, Mitermayer G. Reis, Gregory A. Dasch, Elba R.S. de Lemos, Albert I. Ko

**Affiliations:** Author affiliations: Medicine and Public Health School of Bahia, Salvador, Brazil (N. Silva);; Centers for Disease Control and Prevention, Atlanta, Georgia, USA (M.E. Eremeeva, C.D. Paddock, G.A. Dasch);; Instituto Oswaldo Cruz, Rio de Janeiro, Brazil (T. Rozental, A.R.M. Favacho, E.R.S. de Lemos);; Instituto Oswaldo Cruz, Salvador (G.S. Ribeiro, E.A.G Ramos, M.G. Reis, A.I. Ko);; Federal University of Bahia, Salvador (G.S. Ribeiro);; Yale School of Public Health, New Haven, Connecticut, USA (A.I. Ko)

**Keywords:** Spotted fever group rickettsiosis, rickettsia, eschar, multiple-locus sequence analysis, ticks, molecular diagnosis, dispatch

## Abstract

In Brazil, Brazilian spotted fever was once considered the only tick-borne rickettsial disease. We report eschar-associated rickettsial disease that occurred after a tick bite. The etiologic agent is most related to *Rickettsia parkeri, R. africae, and R. sibirica* and probably widely distributed from São Paulo to Bahia in the Atlantic Forest.

Brazilian spotted fever (BSF), caused by *Rickettsia rickettsii*, was at one time considered the only tick-borne rickettsial disease in Brazil (*1*). Its transmission in 5 southern states is primarily associated with *Amblyomma cajennense, A. aureolatum*, and *Rhipicephalus sanguineus* ticks; however, many other rickettsiae of unknown pathogenicity are carried by ticks in Brazil (*1*,*2*). We describe an eschar-associated rickettsiosis in a traveler from the state of Bahia, Brazil; this disease seems to have been caused by the same *Rickettsia* sp. that caused a similar disease in São Paulo in 2009 (*3*).

## The Case

In April 2007, a 30-year-old man from Bahia sought care for a 6-day febrile illness that began 9 days after he found a tick attached to his right wrist while hiking and camping in the Chapada Diamantina National Park in Paty Valley (12°48′26′′S, 41°19′53′′W), a semiarid region in Bahia. Primary signs and symptoms were fever (39–40°C), severe myalgia, and swelling and pain at the site of the tick bite. Two days after onset of illness, the man noticed a scab forming on his right wrist and painful swelling in his right axillary region, followed 2 days later by a generalized rash and painful ulcerative lesions in the mouth. The patient sought medical care, and an outpatient physician prescribed acetaminophen and cefadroxil, which did not reduce symptoms.

**Figure 1:**
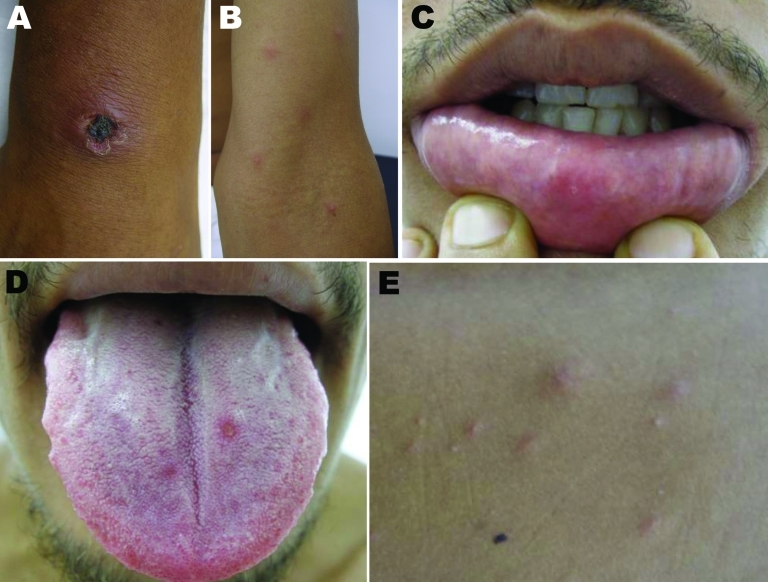
Lesions on day 6 of illness of patient with eschar-associated rickettsial disease, Bahia, Brazil, 2007. A) Eschar on right wrist; B) papular skin rash on left elbow; C) ulcerated lesion on lower lip; D) erosions on tongue mucosa; E) vesicular papular lesions on trunk.

On day 6 of his illness, the patient sought care from an infectious disease specialist, who noted a 2.5-cm eschar on the patient’s wrist (Figure 1, panel A); disseminated papular rash on his face, trunk, and upper extremities (Figure 1, panel B); and several small erosions on his tongue, buccal mucosa, and lips (Figure 1, panels C, D). The mucosal erosions were painful, and some skin papules formed small pustules (Figure 1, panel E). In the right axilla was a tender, enlarged, 3-cm lymph node. Results of a hemogram and blood biochemistry were unremarkable except for a high level (425 U/L) of lactic dehydrogenase. A rickettsial disease was considered, and the patient was given doxycycline (100 mg 2×/d) for 14 days. The fever and generalized rash resolved within 2 days, and the eschar healed completely within 2 weeks after initiation of therapy.

Acute-phase and convalescent-phase serum samples were evaluated by microimmunofluorescence assay for antibodies to spotted fever group rickettsiae (SFGR) (*4*). Before antimicrobial drug therapy was started, biopsy specimens of the papule and the scab from the eschar were collected, preserved in 10% formol, and evaluated by routine histopathology, immunohistochemical staining, and PCR (*4*,*5*).

Serum collected on day 6 of the illness was nonreactive with *R. rickettsii* and *R. parkeri* antigens (class-specific immunoglobulin G [Ig] and IgM <32 for both assays, cutoff >64). Subsequent testing determined IgG/IgM titers on day 12 to be 128/<32 against *R. parkeri* and 128/32 against *R*. *rickettsii* antigens and on day 19 to be 128/64 and 512/32, respectively.

Hematoxylin and eosin–stained sections of the papule biopsy specimen demonstrated lymphohistiocytic perivascular inflammatory cell infiltrates in the superficial to middle dermal layers. Immunohistochemical staining for SFGR showed rare antigens in a few small foci of perivascular inflammation.

**Figure 2:**
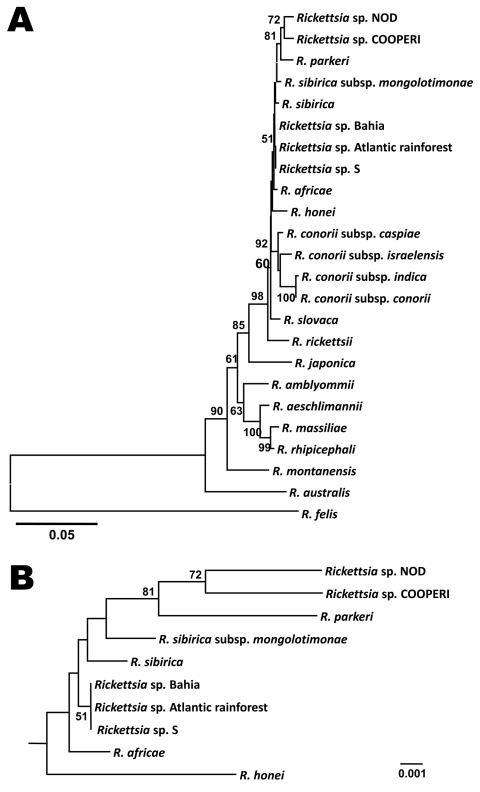
Genetic relationships of the spotted fever group rickettsiae (SFGR) detected in tissue of patient with eschar-associated rickettsial disease, Bahia, Brazil, 2007. Sequence comparison was conducted with MEGA version 4 (www.megasoftware.net). The phylogenetic optimal tree was inferred by using the neighbor-joining method, and distances were evaluated by implementing the Kimura 2-parameter model of substitution (sum of branch length = 0.58588522). In total, 323 nt sites of *glt*A and 401 nt sites of *omp*A were concatenated and evaluated; primer sequences and sites containing gaps and deletions were excluded from the analysis. Statistical reliability of the tree is based on 1,000 bootstrap replicates; only bootstrap values >50 are shown above the branches. The corresponding sequences of reference species and isolates were obtained from the National Center for Biotechnology Information GenBank database. A) Genetic association of *Rickettsia* sp. Bahia and other previously characterized SFGR; B) expanded tree of relationships among new SFGR to *R. africae*, *R. parkeri*, *R. sibirica*, *Rickettsia* sp. S and Atlantic Forest. Scale bars indicate nucleotide substitutions per site.

The sequences for *omp*A (632-bp, GenBank accession no. GQ853063) from the scab and *glt*A (382-bp, GenBank accession no. GQ900666) from the papule specimen each had 100% identity to homologous gene sequences of SFGR detected recently in an eschar specimen from a patient from Peruibe, São Paulo (*3*). The sequences from both organisms were most related to SFGR strain S previously reported from Armenia (*6*) but were not identical to *R. sibirica*, *R*. *parkeri*, and *R. africae* (Figure 2). The nucleotide sequence of a 928-bp *sca*4 fragment (GenBank accession no. GQ853064) had 99% identity to the homologous fragment of *R. parkeri* (GenBank accession no. AF155059), and the conserved 17-kDa protein gene amplicon (GenBank accession no. GQ853062) was similar to those of many SFGR.

## Conclusions

During the past decade, many newly identified tick-borne rickettsiae from South America have been described (*1*,*2*), including *R. parkeri*, *R. massiliae*, *R. amblyommii*, *R. bellii*, and other *Rickettsia* spp. of unknown pathogenicity. We describe another confirmed case of a novel eschar-associated SFGR disease in Brazil.

Development of an eschar is a characteristic manifestation of rickettsioses caused by *R. parkeri,* 364D *Rickettsia*, and *R. massiliae* (*4*,*7*). Possible eschar formation in association with Rocky Mountain spotted fever has been reported (*8*), but this manifestation does not seem to be a hallmark of disease caused by *R. rickettsii* or of other rickettsioses in Brazil and South America (*2*). BSF has been most often confirmed solely by serologic testing; however, atypical clinical manifestations, including eschar formation and lymphadenopathy, have been described (*9–­­­12*). Lymphadenopathy and ulcers on the oral mucosa, as found for this patient, have been found in patients with rickettsiosis caused by *R. parkeri* and African tick bite fever (caused by *R*. *africae*) (*4*,*13*) but not in the index case-patient from São Paulo (*3*), who seemed to have less severe clinical manifestations than the patient described in this report.

In the scientific literature from Brazil, the earliest reference to an eschar in a suspected case of BSF was in 1932 (*12*). Subsequent eschar-associated cases have been identified in regions where BSF is endemic (e.g., the states of Minas Gerais, Rio de Janeiro, and Espirito Santo) (*9*–*11*) and in regions where it is not endemic (e.g., states of Santa Catarina, situated along the Argentina border, and Bahia [*14*], where the case reported in this article occurred). Furthermore, clinical descriptions of eschar-associated rickettsioses in Brazil have been reported from BSF-endemic areas with large populations of *A. dubitatum* ticks but no known *A. triste* ticks, which are recognized vectors of *R. parkeri* in southern Brazil (*15*). Although *A. dubitatum*, a human biting tick that is highly prevalent in many BSF-endemic areas (*2*), is a potential candidate for transmission of *R. parkeri* to humans in Brazil, this tick species and its vertebrate hosts, capybaras, have not yet been described in the Paty Valley, Bahia, where the patient acquired the rickettsial infection. Unfortunately, the ticks causing both cases in São Paulo and Bahia were not available for identification.

The taxonomic status of the etiologic agent of this novel rickettsiosis in Brazil cannot be definitively determined until it is isolated. On the basis of the available genetic information presented here and elsewhere (*3*), the pathogen detected in the cutaneous lesion of the patients from Bahia and São Paulo is equally distant from *R. africae*, *R. parkeri*, and *R. sibirica*. Each of these 3 SFGR is among species long accepted by International Committee of Systematics of Prokaryotes, and this status is consistent with their long evolutionary divergence and differences in their vectors and geographic distributions. Molecular confirmation can and must therefore be used to identify new rickettsial agents because they cannot be identified by clinical case presentations or serologic analyses. Additional efforts will be required to establish the full genetic diversity and range of tick and animal reservoirs of SFGR in Brazil and to determine the prevalence and clinical presentations of different rickettsioses in humans. Clinicians should be alert for tick-borne infectious diseases resulting from ecotourism activities, especially in parks and ecologic reserves in the areas of the Atlantic Forest and other areas of Brazil where many rickettsiae-infected ticks have been identified and most BSF cases have been reported.

## Addendum

Since submission of this article, recent investigation in Brazil has identified *A. ovale* ticks as potential vectors for the spotted fever group *Rickettsia* sp. described here (*16*).

## References

[1] Parola P, Labruna MB, Raoult D. Tick-borne rickettsioses in America: unanswered questions and emerging diseases. Curr Infect Dis Rep. 2009;11:40–50. 10.1007/s11908-009-0007-5 19094824

[2] Labruna MB. Ecology of *Rickettsia* in South America. Ann N Y Acad Sci. 2009;1166:156–66. 10.1111/j.1749-6632.2009.04516.x 19538276

[3] Spolidorio MG, Labruna M, Mantovani E, Brandao P, Richtzenhain L, Yoshinari N. Novel spotted fever group rickettsiosis, Brazil. Emerg Infect Dis. 2010;16:521–3. 10.3201/eid1603.091338 20202436 PMC3322033

[4] Cragun WC, Bartlett BL, Ellis MW, Hoover AZ, Tyring SK, Mendoza N, The expanding spectrum of eschar-associated rickettsioses in the United States. Arch Dermatol. 2010; Epub ahead of print. 10.1001/archdermatol.2010.48 20404224

[5] Eremeeva ME, Bosserman EA, Demma LJ, Zambrano ML, Blau DM, Dasch GA. Isolation and identification of *Rickettsia massiliae* from *Rhipicephalus sanguineus* ticks collected in Arizona. Appl Environ Microbiol. 2006;72:5569–77. 10.1128/AEM.00122-06 16885311 PMC1538723

[6] Eremeeva M, Balayeva N, Roux V, Ignatovich V, Kotsinjan M, Raoult D. Genomic and proteinic characterization of strain S, a rickettsia isolated from *Rhipicephalus sanguineus* ticks in Armenia. J Clin Microbiol. 1995;33:2738–44. 8567916 10.1128/jcm.33.10.2738-2744.1995 PMC228566

[7] Garcia-Garcia JC, Portillo A, Núñez M, Santibáñez S, Castro B, Oteo J. A patient from Argentina infected with *Rickettsia massiliae.* Am J Trop Med Hyg. 2010;82:691–2. 10.4269/ajtmh.2010.09-0662 20348520 PMC2844561

[8] Walker DH, Gay RM, Valdes-Dapena M. The occurrence of eschars in Rocky Mountain spotted fever. J Am Acad Dermatol. 1981;4:571–6. 10.1016/S0190-9622(81)70059-8 7240465

[9] Angerami RN, Resende MR, Feltrin AF, Katz G, Nascimento EM, Stucchi RS, Brazilian spotted fever: a case series from an endemic area in southeastern Brazil: clinical aspects. Ann N Y Acad Sci. 2006;1078:252–4. 10.1196/annals.1374.044 17114716

[10] Costa PSG, Assis RVC, Costa SMCR, Valle LMC, Brigatte ME. Three cases of spotted fever group rickettsiosis with typhus eschar–like lesion (tache noire) reported: species other than *R. rickettsii* at large? Rev Bras Parasitol Vet. 2004;13(Suppl):360.

[11] de Lemos ER, Alvarenga FB, Cintra ML, Ramos MC, Paddock CD, Ferebee TL, Spotted fever in Brazil: a seroepidemiological study and description of clinical cases in an endemic area in the state of São Paulo. Am J Trop Med Hyg. 2001;65:329–34. 11693878 10.4269/ajtmh.2001.65.329

[12] Piza JT. Considerações epidemiológicas e clínicas sobre o tifo exantemático de São Paulo. In: Piza JT, Meyer JR, Gomes LS, editors. Typho exanthematico de São Paulo. São Paulo (Brasil): Sociedade Impressora Paulista; 1932. p. 11–119.

[13] Jensenius M, Fournier P-E, Vene S, Hoel T, Hasle G, Henriksen AZ, African tick bite fever in travelers to rural sub-equatorial Africa. Clin Infect Dis. 2003;36:1411–7. 10.1086/375083 12766836

[14] Plank SJ, Teixeira RS, Milanesi ML. Febre maculosa em Salvador: descrição de um caso. Rev Med Bahia (Salvador). 1979;25:330–4.

[15] Silveira I, Pacheco RC, Szabó MPJ, Ramos HGC, Labruna MB. First report of *Rickettsia parkeri* in Brazil. Emerg Infect Dis. 2007;13:1111–3. 18214195 10.3201/eid1307.061397 PMC2878225

[16] Sabatini GS, Pinter A, Nieri-Bastos FA, Marcili A, Labruna MB. Survey of ticks (Acari: Ixodidae) and their rickettsia in an Atlantic rain forest reserve in the State of São Paulo, Brazil. J Med Entomol. 2010;47:913–6. 10.1603/ME10073 20939390

